# Habitat Disturbances Modulate the Barrier Effect of Resident Soil Microbiota on *Listeria monocytogenes* Invasion Success

**DOI:** 10.3389/fmicb.2020.00927

**Published:** 2020-05-28

**Authors:** Aymé Spor, Angela Rocio Ortiz Camargo, David Bru, Sabrina Gaba, Dominique Garmyn, Laurent Gal, Pascal Piveteau

**Affiliations:** ^1^Agroécologie, AgroSup Dijon, INRAE, Université de Bourgogne, Université de Bourgogne Franche-Comté, Dijon, France; ^2^USC 1339, Centre d’Études Biologiques de Chizé, INRAE, Villiers-en-Bois, France; ^3^CNRS, UMR 7372 Centre d’Études Biologiques de Chizé, Université de La Rochelle, La Rochelle, France; ^4^UR OPAALE, INRAE, Rennes, France

**Keywords:** environmental disturbances, Listeria monocytogenes, invasion, microbial diversity, community membership

## Abstract

Microbial communities are continuously exposed to the arrival of alien species. In complex environments such as soil, the success of invasion depends on the characteristics of the habitat, especially the diversity and structure of the residing bacterial communities. While most data available on microbial invasion relies on experiments run under constant conditions, the fate of invading species when the habitat faces disturbances has not yet been addressed. Here, we designed experiments to assess the consequences of habitat disturbance on the success of ongoing microbial invasion. We investigated (i) if disturbance-induced alterations in resident microbial communities could mitigate or facilitate invasion of *Listeria monocytogenes*, (ii) if disturbance itself could either improve or reduce the invader’s fitness and (iii) if the invading species alters the structure of indigenous microbial communities. Our data show that environmental disturbances affect invasion patterns of *L. monocytogenes* in soils. Intriguingly, successful invasion was recorded in a regimen of disturbances that triggered small changes in microbial community structure while maintaining high bacterial diversity. On the opposite, dramatic decline of the invader was recorded when disturbance resulted in emergence of specific communities albeit concomitant with a diversity loss. This suggests that community composition is more important than its diversity when it comes to prevent the establishment of an invading species. Finally, shifts in bacterial communities during the disturbance event were strengthened by the presence of the invader indicating a major impact of invasion on microbial diversity when the habitat faces disturbance.

## Introduction

Biological invasion is the process by which alien species establish and colonize niches. This aspect of the ecology of plants and animals has been addressed for decades, while microbial invasion is a developing field (Kinnunen et al., 2016). Biological invasion is a major process affecting ecosystem functioning (Ehrenfeld, 2010). In soil, establishment of an invading alien bacterial species depends on resident community assembly. Experimental evidences suggest that both a loss of diversity and shifts in bacterial community composition facilitate invasion (van Elsas et al., 2012; Vivant et al., 2013a; Yao et al., 2014; Chapelle et al., 2016). This indicates that high bacterial species richness increases the resistance to invasion, but that the effect is modulated by the nature of the species in the community, some of which may act as invader repellent. Furthermore, the arrival of alien bacteria in a resident community can induce changes in the diversity and functioning of resident communities (Yao et al., 2014; Chapelle et al., 2016; Mallon et al., 2018). Bacterial competition for resources is a key factor affecting the resistance of the resident community to invasion (Wei et al., 2015) and hence strongly affects the establishment of alien bacteria in soils (Ma et al., 2015).

Beside biotic factors, environmental disturbances are strong drivers of soil microbial communities over time. Changes in microbial communities vary with the type and the intensity of the disturbance. These changes can eventually leave a breach for the invasion of an alien species when for example (*i*) niches were vacant due to the environmental change, or (*ii*) competitors of this alien species were removed after this change. Disturbances induced by variation in temperature such as freeze-thaw or heat, are of particular interest when considering soil microbial communities because they can drastically impact ecosystem functioning and cause long-term shifts in community diversity and structure (Griffiths and Philippot, 2013; Calderon et al., 2018). Interestingly, as a consequence of heat treatments of soil microcosms, the subsequent invasion of *Pseudomonas fluorescens*, *Escherichia coli*, *Ralstonia solanacearum*, and *Bacillus amyloliquefaciens* was facilitated due to loss of resident community diversity and increased resource availability (Liu et al., 2012; Ma et al., 2015). In the context of climate change, extreme temperature events acting as disturbances are expected to occur more frequently and invasions are expected to increase. Other factors such as drought result in similar disturbances. To our knowledge, the fate of alien bacteria facing a disturbance event during invasion, and the overall consequences on the success of invasion, have not yet been addressed.

Here, we designed experiments under controlled conditions to test for three hypotheses related to how habitat disturbances may affect the fate of an alien bacterial species during invasion and the overall invasion success:

Hypothesis 1: disturbance-induced changes in resident microbial communities alter alien’s fitness;

Hypothesis 2: disturbance-induced changes in the physiology of the invading species either improve or reduce its fitness in natural communities.

Hypothesis 3: the invading species alters the structure of indigenous microbial communities.

Soil microcosms were used as model habitats and *Listeria monocytogenes* was selected as the alien invasive microorganism. Indeed *L. monocytogenes* is a versatile bacterial species able to colonize numerous habitats including water systems and sediments, waste water treatment plants, vegetation, soil, animals, food factories, and foodstuff (Ivanek et al., 2006; Vivant et al., 2013b; Ricci et al., 2018). It has the ability to grow at temperatures as low as 2°C and as high as 42°C. It is one of the most resistant bacteria to heat treatment among non-spore forming bacteria (Doyle et al., 2001). It is also the agent of listeriosis, a life-threatening disease (Radoshevich and Cossart, 2018). Its striking ability to adapt its physiology to a wide range of environmental conditions is supported by the characteristics of its genome. Indeed a large proportion of the genome is dedicated to regulation and to the transport and use of a wide range of resources (Glaser et al., 2001; Fratamico et al., 2011). Its versatility has been shown in numerous studies, as illustrated by the fact that its incubation in lagoon and soil environments triggers major transcriptomic shifts (Piveteau et al., 2011; Vivant et al., 2015, 2017). Although considered as a telluric microorganism, the ability of *L. monocytogenes* to establish in soil depends on abiotic and biotic edaphic characteristics (Locatelli et al., 2013; Vivant et al., 2013a). The Basic Cation Saturation ratio, as well as clay content, were shown to be correlated to its survival in soil (Locatelli et al., 2013). Experimental evidences also suggest that the diversity and composition of the soil microbiota are key factors affecting its survival in soil (Vivant et al., 2013a).

In this paper, in order to investigate how disturbances induced by extreme temperature shifts affect the success of ongoing alien bacterial invasion, we compared the fate of *L. monocytogenes* in soil microcosms subjected or not to successive disturbances to highlight the determinants of its invasion success under ongoing environmental variations. Temperature shifts were chosen to simulate environmental disturbances. Sterile soil was included in the experimental design in order to disconnect direct effects of the disturbance on the population of *L. monocytogenes* from indirect effects due to possible shifts in indigenous microbiota. The invasion success was estimated as the ability of *L. monocytogenes* to establish and persist in soil. We then examined if the persistence of *L. monocytogenes* in disturbed soil was related to changes in the resident soil bacterial communities induced by disturbances, or by the intrinsic physiological adaptation of *L. monocytogenes* to abiotic changes in its environment, i.e., changes in temperature, in soil properties or both.

## Materials And Methods

### Invader Bacterial Strain and Inoculum Preparation

*Listeria monocytogenes* L9, a rifampicin resistant derivative of the type strain *L. monocytogenes* EGD-e (Lemunier et al., 2005) was used as invading species. Frozen stock cultures were stored at −70°C. A first overnight culture of 10 ml of Tryptone Soy Broth (TSB; Conda, Spain) was prepared from the stock culture and incubated at 25°C. This culture was transferred in fresh TSB (1% v/v) and incubated 24 h at 25°C. Grown cultures were centrifuged (10000 *g*, 10 min, room temperature) and the pellets suspended in sterile distilled water.

### Soil Microcosms

Two different soils were used in this study. Soil E [Époisses, France (47° 30′ 22.1832′ N, 4° 10′ 26.4648′′ E)] is a clay loamy soil with a pH of 7.15. In contrast, soil D [Dompierre-en-Morvan, France (47° 23′ 47.677′′ N, 4° 23′ 49.2744′ E)] is an acidic loamy sandy soil (pH 5.46). In each site, three areas 20 m apart from each other were located. In each area, five soil cores (0–20 cm) were sampled and pooled in a composite sample. Soils were sieved to 0.4 mm. *L. monocytogenes* was not detected in these two composite samples. Aliquots of both soils were sterilized by γ-radiation (45 kGy; Ionisos, Dagneux, France). Sterilized and non-sterile soil microcosms of each soil were prepared. Sterile soils allow investigating the sole effect of disturbances, while non-sterile conditions were used to explore the effect of soil community diversity and composition on the invasion success of *L. monocytogenes* in the presence and absence of disturbances.

### Experimental Designs

**FIGURE 1:**
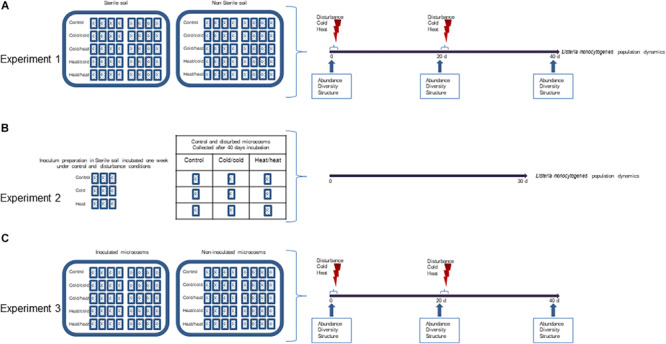
Experimental design. Three sets of experiments were conducted. **(A)** To test the effect of disturbances on the invasion success, microcosms were prepared with two types of soil E and D, either sterilized by γ-irradiation or non-sterilized; after inoculation, microcosms were incubated for 40 days at 20°C (control) or submitted to disturbance by shifting the temperature to –20°C (cold) and/or + 42°C (heat) at day 0 and day 20; *Listeria monocytogenes* was enumerated periodically throughout the incubation period. Samples were collected for DNA extraction and diversity analysis at time 0, 20, 40 days. **(B)** To test the hypothesis that disturbances may prime intrinsic factors facilitating invasion, inoculums were prepared by incubating *L. monocytogenes* for 1 week in sterile soil undergoing disturbance (cold, heat) or kept undisturbed at 20°C; 40 days old disturbed or undisturbed microcosms were then seeded with these conditioned soil inoculums. **(C)** Microcosms were prepared with two types of non-sterile soil E and D; one set of microcosms was inoculation with *L. monocytogenes* while the other set was kept non-inoculated; microcosms were incubated for 40 days at 20°C (control) or submitted to disturbance by shifting the temperature either to –20°C (cold/cold) or + 42°C (heat/heat) at day 0 and day 20. After DNA extraction, the diversity and community structure of the soil microbiota was analyzed.

Three experiments were designed to address the three working hypotheses (Figure 1).

#### Experiment 1: Effect of Temperature Disturbance Sequences on the Fate of *L. monocytogenes* in Soil

This experiment addressed two questions: (*i*) how temperature disturbances affect the invasion success of *L. monocytogenes* and (*ii*) how the presence of endogenous soil bacterial communities modulates these effects. Therefore, in order to untangle direct effects of temperature shifts and indirect consequences of disturbances through alterations of the biotic environment, experiments were performed in sterilized and non-sterile soil microcosms. Soil microcosms were prepared in 500 ml plasma flasks with 150 g of soil coming from the two different locations. At the beginning of the experiment (T0), microcosms were inoculated with the equivalent of 10^8^ *L. monocytogenes* CFU/g dry soil in the adequate water volume to reach 60% of the water holding capacity (WHC). After inoculation, microcosms were closed with sterile lids and population dynamics were followed for 45 days. Two environmental disturbances were considered: a freeze-thaw disturbance (−20°C) referred to ‘cold’ and a heat disturbance (+ 42°C) referred to ‘heat.’ The duration of each treatment was twice 30 h separated by 24 h at 20°C. The disturbances were applied at the start of the experiment (T0) and after 20 days of incubation (T20). For each soil, four disturbance sequences were applied in sterilized and non-sterile soil microcosms: Cold_T__0_/Cold_T__20_, Cold_T__0_/Heat_T__20_, Heat_T__0_/Cold_T__20_ and Heat_T__0_/Heat_T__20_. Control undisturbed sterilized and non-sterile soil microcosms were incubated at the constant temperature of 20°C for the total duration of the experiment. The soil moisture was checked after treatments and during incubation. The water content was adjusted if required. In this experiment *L. monocytogenes* was numerated throughout the duration of incubation. Soil DNA samples were collected at T0, T20 and T40. In this experiment, four biological replicates were analyzed.

#### Experiment 2: Soil Acclimatization Experiment

The acclimatization experiment aimed to decipher whether or not temperature shifts could trigger the onset of intrinsic mechanisms that could possibly confer physiological adaptation of *L. monocytogenes*, hence improved survival in disturbed soils. For this experiment, we focused only on soil E because it was the soil in which *L. monocytogenes* displayed the longest persistence. Inoculums were prepared in sterile soil microcosms (60% WHC) as follows: a 24 h TSB culture at 25°C was centrifuged (10000 *g*, 10 min, room temperature) and the pellets suspended in sterile distilled water. Fifteen g of sterile soil microcosms were inoculated with the equivalent of 10^6^ *L. monocytogenes* CFU/g dry soil in the adequate water volume to reach 60% of the WHC. After inoculation, these microcosms underwent either a cold, heat or no disturbance at all. The duration of each treatment was twice 30 h separated by 24 h at 20°C. Inoculums were collected after 1 week of incubation. These microcosms were used as pre-treated soil inoculums for the acclimatization experiment.

Secondly, non-inoculated non-sterile soil microcosms were prepared in 500 ml plasma flasks with 150 g of soil E. Microcosms were then treated with either Cold_T__0_/Cold_T__20_, Heat_T__0_/Heat_T__20_ or no disturbance at all, as described in experiment 1. At the end of the 40 days incubation period, these disturbed microcosms were aliquoted in nine microcosms of 50 g, the water content was checked and if required adjusted to 60% of the WHC.

The pre-treated soil inoculums were used to seed the non-sterile disturbed and control microcosms with the equivalent of 10^6^ *L. monocytogenes* CFU/g dry soil. Inoculated microcosms were then incubated for 30 days at the constant temperature of 20°C. The population of *L. monocytogenes* was quantified regularly over this incubation period. This experiment was performed in triplicates.

#### Experiment 3: Investigation of the Effect of Inoculating *L. monocytogenes* on Bacterial Community Composition During and After Disturbance

Finally, the third experiment examined how *L. monocytogenes* invasion affects the structure of the resident bacterial communities, during and after different disturbance sequences. Non-sterile soil microcosms were prepared as described above and split into two sets. One set of microcosms was inoculated with *L. monocytogenes* at the same concentration and same water content as in experiment 1, and the other one was kept non-inoculated. The water content of non-inoculated microcosms was adjusted similarly with sterile distilled water. After inoculation of the first set, all microcosms were closed with sterile lids and incubated at 20°C for 45 days except during the periods of treatment as detailed in experiment 1. Only two disturbance sequences were considered in experiment 3: Cold_T__0_/Cold_T__20_ and Heat_T__0_/Heat_T__20_. Control undisturbed microcosms were incubated at the constant temperature of 20°C. Soil DNA samples were collected at T0, T20 and T40. In this experiment, four biological replicates were analyzed.

### Estimation of *L. monocytogenes* Population Size

In order to enumerate *L. monocytogenes* populations, 1 g equivalent dry weight of each microcosm was added to 9 ml tryptone salt and 0.7 g glass beads (Sigma, France). Microorganisms were suspended by agitation with a vortex operated full speed for 2 min. The soil slurry was serially diluted and plated onto PALCAM plates supplemented with 0.1 g/L rifampicin and 0.1 g/L cycloheximide. When required, pour plating was used for enumeration in order to improve sensitivity. The limit of detection of the assay was 10 CFU/g dry soil.

### Soil DNA Extraction and 16S rDNA Gene MiSeq Sequencing

Total DNA was extracted from 250 mg dry soil with Fast DNA spin kit (MP Bio, France) according to the manufacturer’s manual. DNA’s integrity was assessed after electrophoresis on 1% agarose gel. Total DNA concentration was quantified by fluorometry using a Quant-iT PicoGreen dsDNA Assay Kit (Invitrogen, Cergy-Pontoise, France) following the manufacturer’s instructions.

A two-step PCR procedure was used for library preparation. First of all the V3–V4 hypervariable region of bacterial 16S rDNA gene was PCR amplified with primers Pro341F (5′-TCGTCGGCAGCGTCAGATGTGTATAAGAGACAGNNNNC CTACGGGNBGCASCAG-3′) and Pro805R (5′-GTCTCGTGG GCTCGGAGATGTGTATAAGAGACAGNNNNGACTACNVG GGTATCTAATCC-3′) (Takahashi et al., 2014). Duplicate 15 μL amplification reactions were prepared with 7.5 μL Phusion High-Fidelity PCR Master Mix (Thermo Fischer Scientific), 0.25 μM of each primer, 250 ng T4 gp32 (MPBio) and 1 ng template DNA. After 3 min at 98°C, amplification proceeded during 25 cycles of 98°C for 30 s, 55°C for 30 s, and 72°C for 30 s, with a final extension at 72°C for 10 min. PCR products were pooled before the second PCR reaction in which multiplexing index sequences were added. This second PCR reaction was done in duplicate. A unique multiplex primer pair combination was used for each sample. The 30 μL reaction mixture was composed of 15 μL Phusion High-Fidelity PCR Master Mix (Thermo Fischer Scientific), 250 ng T4 gp32 (MPBio), 1 μM of one forward and one reverse multiplex primer and 6 μL of first step PCR product. Thermal cycling conditions were as for the first reaction except that the reaction was stopped after eight cycles. PCR products were pooled, checked on 1% agarose gel, cleaned-up, and purified. Quantified products were normalized with SequalPrep Normalization plates kit (Thermo Fisher Scientific) and pooled. These 203 samples were sent for sequencing on MiSeq (Illumina, 2 bp × 250 bp, MiSeq reagent kit v2, 500 cycles). Sequences were processed with Illumina MiSeq Reporter software (version 2.5.1.3) for demultiplexing and trimming of adaptors and barcodes.

### Bioinformatics Analysis of 16S rRNA Gene rDNA Diversity

The sequence data were analyzed using an in-house developed Jupyter Notebooks (Kluyver et al., 2016) piping together different bioinformatics tools. Briefly, R1 and R2 sequences were assembled using PEAR (Zhang et al., 2014) with default settings. Further quality checks were conducted using the QIIME pipeline (Caporaso et al., 2010a) and short sequences were removed (<400 bp). Reference based and *de novo* chimera detection, as well as clustering in OTUs were performed using VSEARCH (Rognes et al., 2016) and the adequate reference databases (Greengenes’ representative set of sequences). The identity thresholds were set at 94% for 16S rRNA gene data based on replicate sequencing of a bacterial mock community containing 40 bacterial species. Representative sequences for each OTU were aligned using PyNAST (Caporaso et al., 2010b) and a 16S rRNA gene phylogenetic tree was constructed using FastTree (Price et al., 2010). Taxonomy was assigned using UCLUST (Edgar, 2010) and the latest released greengenes database (v.05/2013, McDonald et al., 2012). Sequences were deposited to the SRA at NCBI under the accession number PRJNA506131.

We used three diversity metrics to describe the structure of microbial communities: the Faith’s Phylogenetic Diversity (Faith, 1992), species richness (observed species) and evenness (Simpson’s reciprocal index, equitability) were calculated based on rarefied OTU tables (3500 and 5500 sequences per sample for experiment 1 and 2, respectively). UniFrac distance matrices (Lozupone and Knight, 2005; Lozupone et al., 2011) were also computed to detect global variations in the composition of microbial communities.

### Statistical Analyses

The fate of *L. monocytogenes* invasion was modeled using a Cox regression as a function of treatment (four levels), the type of soils (two levels) and the presence of microbiota (two levels) on the fate, as well as their two-way interactions. For each time point, we further analyzed the difference in *L. monocytogenes* population per soil type and in presence of absence of bacteria between the treatment modalities using analyses of variance (ANOVA).

The effect of the explaining variables (Treatment, type of Soils, Inoculation, Timepoint) on the responses variables (*L. monocytogenes* CFUs, diversity indices, discriminant OTUs) were assessed using ANOVAs followed by a Tukey test. The FDR procedure was used to correct for multiple testing when detecting discriminant OTUs.

All analyses were performed using the software R (version 3.6.0) and the Cox regression with the package survival (Therneau, 2020).

## Results

### Dynamics of *L. monocytogenes* Growth and Survival in Sterile and Non-sterile Soil Microcosms. Experiment 1

**FIGURE 2:**
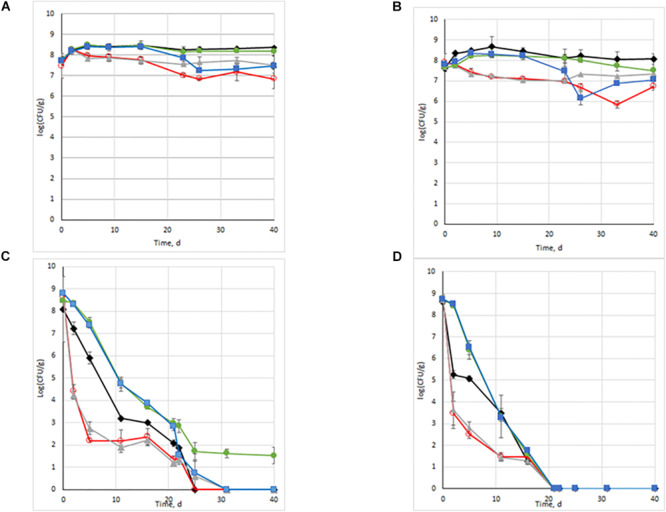
Persistence dynamics of *Listeria monocytogenes* populations in control and in microcosms undergoing disturbances. **(A)** sterilised soil E microcosms; **(B)** sterilised soil D microcosms; **(C)** non sterile soil E microcosms; **(D)** non sterile soil D microcosms. 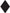: Control; 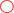: Heat_T0_/Heat_T20_; 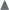: Heat_T0_/Cold_T20_; 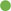: Cold_T0_/Cold_T20_; 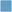: Cold_T0_/Heat_T20_. Time points at which differences between treatments are significant (Tukey test, *P* < 0.05) can be found in Supplementary Data Sheet S1.

In order to investigate the fate of an invading species when habitats undergo disturbances, population dynamics of *L. monocytogenes* were followed over time (Figure 2). In the absence of any endogenous microbial community (sterilized soil) and without disturbance, *L. monocytogenes* successfully invaded the soil, suggesting that the resources required for growth were available (Figures 2A,B). After growth during the first 5 days, the population remained stable until the end of the experiments (45 days of incubation) in both soils. When these microcosms underwent two successive disturbances related to abrupt change in temperature either toward high or low temperature, we observed a significant effect of the type of treatment with similar trends in the two soils (Figures 2A,B and Supplementary Table S1). Early after the start of the experiment in sterilized soils (T2 or T5), two trends were observed differentiating the control, the Cold_T__0_/Cold_T__20_ and Cold_T__0_/Heat_T__20_ treatments from the two treatments starting with the heat treatment (Heat_T__0_/Heat_T__20_ and Heat_T__0_/Cold_T__20_) in which *L. monocytogenes* populations were significantly lower. The Cold_T__0_/Cold_T__20_ treatment did not affect the quantity of *L. monocytogenes* detected during the course of the experiment while the Heat_T__0_/Heat_T__20_ treatment resulted in a significant decrease of the population compared to the control from day 5 until the end of the experiment (Figures 2A,B). When combining the heat and the freezing disturbances, we observed that it is the heat disturbance in the sequence that caused a decrease in *L. monocytogenes* population sizes compared to control. A maximum 10-fold decrease was observed for the heat/heat treatment, but *L. monocytogenes* remained at high abundance levels over the whole incubation period in γ -irradiated soil. These data indicated that, whatever the disturbance regimen, in the absence of any endogenous microbiota a clear colonization of the two soils was observed.

We then assessed how the presence of endogenous soil bacterial communities would modulate these effects. The population of *L. monocytogenes* decreased in all non-sterile soil microcosms (Figures 2C,D). The statistical analysis of survival data identified the presence of the soil community as the most significant factor affecting *L. monocytogenes* abundance (c^2^ = 143.87, df = 1, *P* < 0.0001). In non-sterile soil control microcosms, *L. monocytogenes* failed to invade (Figures 1C,D), and extinction was faster in soil D than in soil E (type of soil x presence/absence of soil community: c^2^ = 25.33, df = 1, *P* < 0.0001). Indeed, the soil type had a significant effect on the fate of the invasion, even in absence of bacteria as *L. monocytogenes* had a lower concentration in soil D than in soil E (soil type: c^2^ = 21.96, df = 1, *P* < 0.0001). Contrary to our expectation, the Cox regression did not evidence a significant effect of the temperature treatments (c^2^ = 3.42, df = 4, *P* = 0.49), revealing overall similar patterns of *L. monocytogenes* population among treatments, but when soil microcosms underwent temperature shifts, results depended on the disturbance sequence used. When comparing *L. monocytogenes* concentrations at each time point, we did observe significant differences in concentrations between experimental treatments in both soils and in presence/absence of microbiota (Figure 2). *L. monocytogenes* concentrations were higher in the treatments starting with cold temperature than in the control, the control being higher than the treatments starting with high temperature (Figure 2). In soil E, a significantly steeper decrease was observed when the temperature initially rose to 42°C (Heat_T__0_/Heat_T__20_ and Heat_T__0_/Cold_T__20_) than in the other conditions. Another steep decrease was evidenced after the second event of temperature rise and *L. monocytogenes* population was no longer detected after 25 days. On the opposite, the initial freezing step (Cold_T__0_/Cold_T__20_ and Cold_T__0_/Heat_T__20_ treatments) resulted in better survival of *L. monocytogenes* population with significantly higher estimates than in control microcosms. After the second freezing disturbance, the population was still significantly higher in the Cold_T__0_/Cold_T__20_ treatment than in control microcosms and remained so until the end of the experiment at approximately 30 CFU/g soil. Note that for the Heat_T__0_/Cold_T__20_ and the Cold_T__0_/Heat_T__20_ treatments, the shape of the decrease in population sizes of *L. monocytogenes* was distinct, but in both cases *L. monocytogenes* was undetectable after 30 days indicating that whatever its chronological order, the heat disturbance blocked *L. monocytogenes* invasion. In soil D microcosms, similar trends were observed but *L. monocytogenes* population decreased faster and could not be detected under any experimental condition after only 3 weeks of incubation. Still, significantly higher *L. monocytogenes* populations were numerated in Cold_T__0_ than in Heat_T__0_ and control within the first 12 days of incubation.

Altogether we showed that disturbances impacted survival of the invading species and, in some cases, habitat disturbances could lead to its persistence.

### Effect of Preadapting *L. monocytogenes* in Sterile Soil on Invasive Potential. Experiment 2

Pre-treatment of the invader population to a given disturbance did not improve its invasiveness in non-sterile microcosms previously disturbed (Supplementary Figure S1). These results therefore showed that the invasive success of *L. monocytogenes* observed during specific habitat disturbance could not be simply ascribed to intrinsic mechanisms of physiological adaptation triggered by the temperature treatment and suggested that changes in extrinsic factors did affect the fate of *L. monocytogenes*.

### Analysis of Soil Microbiota Diversity and Community Structure During Environmental Disturbances. Experiment 1

**FIGURE 3:**
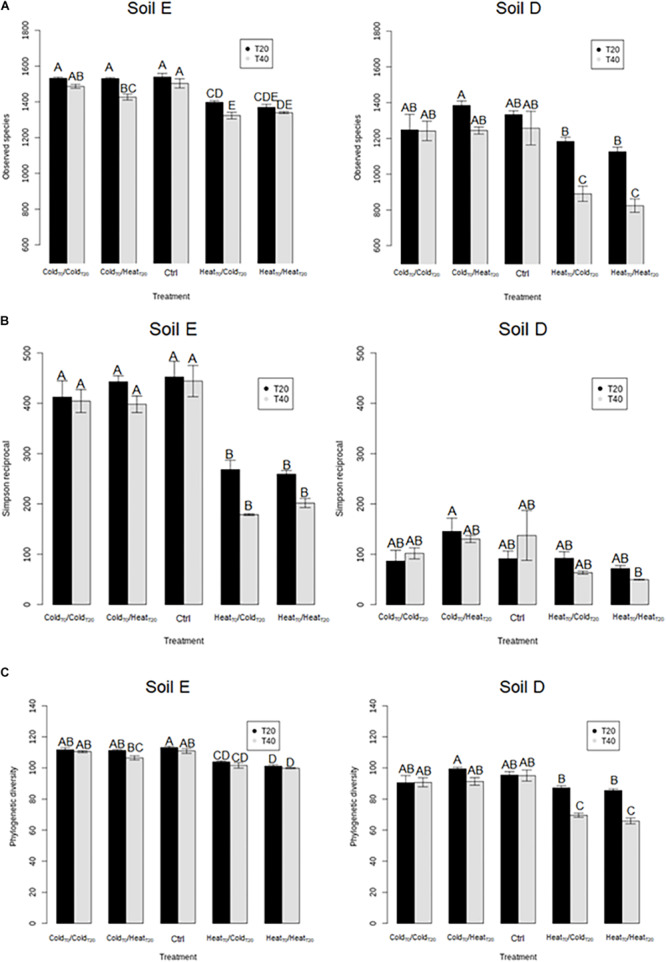
Assessment of bacterial diversity in soil microcosms of soil E and soil D facing ongoing invasion and under a regimen of disturbance. **(A)** Species richness estimated by the observed species index. **(B)** Evenness estimated by Simpson’s reciprocal index and **(C)** Phylogenetic diversity estimated by Faith’s phylogenetic diversity index. Results for sampling at 20 (T20) and 40 (T40) days are shown in black and gray. Letters above the bars indicate the significance of the differences according to Tukey’s test (*P* < 0.05).

**FIGURE 4:**
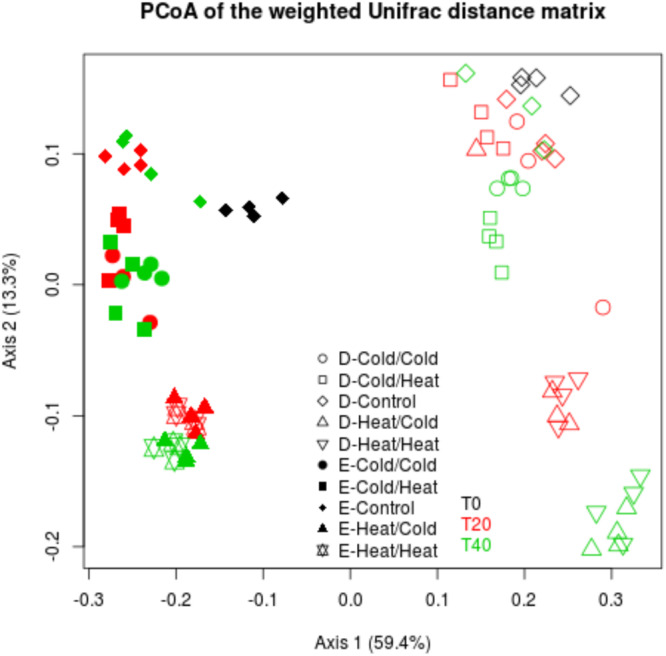
Disturbance treatments affect bacterial community composition in soil E and soil D microcosms facing ongoing invasion and under a regimen of disturbance. Differences in community composition were estimated using the weighted UniFrac distance. This figure represents a PcoA of the weighted UniFrac distance matrix. Black, red and green colors correspond respectively to T0, T20, and T40 samples. Empty and filled symbols correspond to soil D and soil E, respectively.

This prompted us to assess, over the course of the invasion, the overall diversity of the two soil microbiota before (T0), during (T20) and at the end of disturbance sequences (T40) (Figures 3, 4). First of all, the diversity levels observed in the two soils at T0 were comparable with equivalent richness and evenness quantified by the observed species, Simpson reciprocal and equitability estimates. The phylogenetic diversity was slightly but significantly higher in soil E (99.1 ± 1.8) compared to soil D (91.1 ± 1.6) (Tukey test, *P* < 0.05). The composition of the two initial microbial communities was however very different at T0 (Figure 4). The different treatments had various effects on bacterial diversity depending on the soil type and the chronological sequence of disturbances (Figure 3). First, the Heat_T__0_/Heat_T__20_ and Heat_T__0_/Cold_T__20_ treatments had the strongest impact on species richness, phylogenetic diversity and evenness of the bacterial communities in both soils (Figure 3 and Supplementary Figure S2). On the opposite, the Cold_T__0_/Cold_T__20_ treatment had no significant impact on the bacterial diversity, as no differences were found with the control microcosms of both soils.

Interestingly, in the two soils, the Cold_T__0_/Heat_T__20_ and Heat_T__0_/Cold_T__20_ treatments affected differently the bacterial diversity. Indeed, the Heat_T__0_/Cold_T__20_ treatment impacted strongly bacterial diversity with significantly lower estimates than in control microcosms while the Cold_T__0_/Heat_T__20_ treatment did not significantly affect the four measures of diversity in both soils. Results were largely similar when considering bacterial community structures. As evidenced on the projection of the diversity data, the most impacting disturbance sequences were, for the two soils, the Heat_T__0_/Cold_T__20_ and the Heat_T__0_/Heat_T__20_ treatments at both T20 and T40 (Figure 4). The Cold_T__0_/Cold_T__20_ and Cold_T__0_/Heat_T__20_ treatments had only mild effects on bacterial community compositions at both time points compared to control microcosms indicating that both communities were, to some extent, resistant to freeze-thaw.

### Correlation Between Soil Microbiota Diversity and Competitiveness of *L. monocytogenes*. Experiment 1

Interestingly, *L. monocytogenes* persistence was higher in the cold-disturbed microcosms, the habitats showing the highest diversity estimates and the most stable bacterial community composition, which is in discordance with the hypothesis stating that higher diversity may act as a barrier effect to invasion (van Elsas et al., 2012; Vivant et al., 2013a). More so than diversity itself, community membership may be more important when studying resistance to invasion as proposed previously (Vivant et al., 2013a).

Therefore, considering the two extreme treatments (Cold_T__0_/Cold_T__20_ and Heat_T__0_/Heat_T__20_) in terms of survival of *L. monocytogenes* in soil E, we searched for OTUs displaying differential abundances between these two treatments and behaving potentially as invader repellents or facilitators of *L. monocytogenes* survival in this soil (Supplementary Figure S3, S4). Several OTUs were enriched in the Heat_T__0_/Heat_T__20_ treatment compared to the Cold_T__0_/Cold_T__20_ one. Those OTUs belonged to the Actinobacteria (*Streptomyces* OTU), Firmicutes (*Alicyclobacillus* and *Brevibacillus* OTUs) phyla and to the α- (Balneimonas and Kaistobacter OTUs) and δ-proteobacteria (Haliangiaceae) classes. On the other hand, data were analyzed to identify OTUs significantly more abundant after cold treatment than after heat treatment. Such OTUs could be possible invader facilitator candidates, facilitating *L. monocytogenes* persistence via modifications of the soil habitat. Several OTUs were enriched in the Cold_T__0_/Cold_T__20_ treatment compared to the Heat_T__0_/Heat_T__20_ one. They were identified among Acidobacteria (three OTUS), Actinobacteria (Acidimicrobiales, Micrococcaceae, and Nocardioides OTUs), Bacteroidetes (Chitinophagaceae, Cytophagaceae, Flavobacterium and Saprospiraceae OTUs), Fibrobacteres (Fibrobacteria OTU), and α-proteobacteria (Bradyrhizobiaceaea, Devosia and Rhizobium OTUs). However, their abundance was similar in control and Cold_T__0_/Cold_T__20_ treated microcosms. This suggests that these OTUs displaying similar trends as *L. monocytogenes*, might be affected by the temperature rise to 42°C rather than being resistant to freeze-thaw. It is therefore difficult to consider a possible role of these OTUs in facilitating the success of the invasive species in cold-disturbed habitats.

### Effect of *L. monocytogenes* Invasion Combined With Environmental Perturbation on Soil Microbiota Diversity. Experiment 3

**FIGURE 5:**
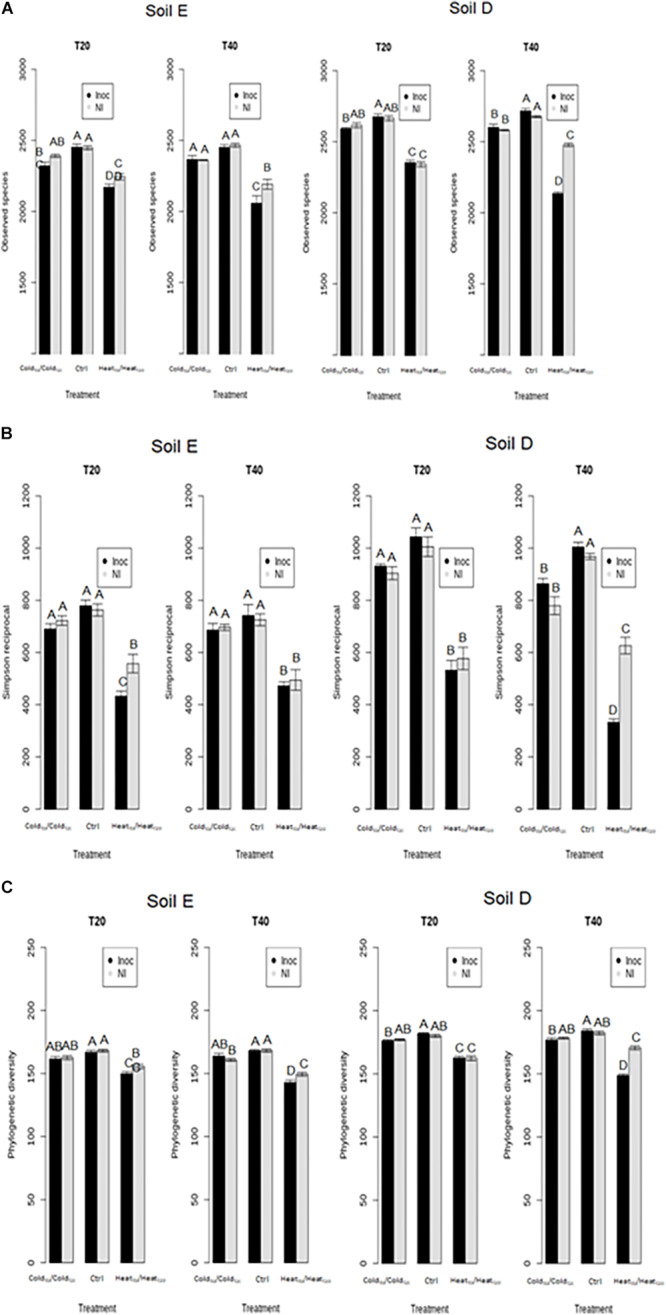
Assessment of the effect of the invasive species on bacterial diversity components of soil E and soil D microcosms facing different disturbance sequences. **(A)** Richness estimated by the observed species index. **(B)** Evenness estimated by Simpson’s reciprocal index. **(C)** Phylogenetic diversity estimated by Faith’s phylogenetic diversity index. Letters above the bars indicate significance of the differences according to Tukey’s test (*P* < 0.05).

The comparison of the diversity of non-inoculated and inoculated control microcosms showed that inoculation of *L. monocytogenes* had a strong effect on the richness, the evenness and the phylogenetic diversity of the bacterial communities of both soils at T0 (Supplementary Figure S5). However, differences in α-diversity estimates between inoculated and non-inoculated control microcosms disappeared at T20 and T40 (Figure 5 and Supplementary Figure S2). This indicates that, in the absence of disturbance, the impact of *L. monocytogenes* on bacterial diversity was transient in both soils and their bacterial communities were able to recover.

**FIGURE 6:**
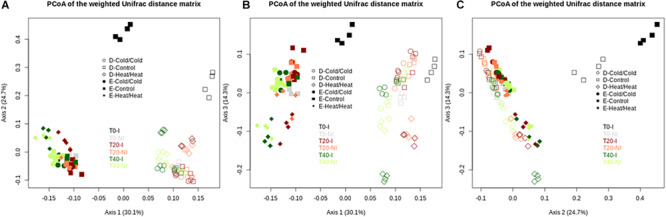
Assessment of the effect of the invasive species on bacterial community compositions of soil E and soil D microcosms facing different disturbance sequences. Differences in community composition were estimated using the weighted UniFrac distance. Projections on PcoA axes 1 to 3 are represented on this figure **(A–C)**. Empty and filled symbols correspond to soil D and soil E, respectively. “I” inoculated microcosms; “NI” non-inoculated microcosms.

During disturbances, the Heat_T__0_/Heat_T__20_ treatment had the strongest effect on α-diversity estimates, with significantly lower values than in the control and cold-treated microcosms (Figure 5 and Supplementary Figure S2). In comparison to controls, Heat_T__0_/Heat_T__20_ treatment significantly affected bacterial community compositions as well (Figure 6). Upon invasion by *L. monocytogenes*, significantly lower α-diversity estimates were recorded compared to non-inoculated ones, for both soils either at T20 or at T40 during this disturbance sequence. We also observed changes in bacterial community composition between inoculated and non-inoculated Heat-disturbed microcosms, especially at both T20 and T40 in soil E and mostly at T40 in soil D (Figure 6). This indicates that arrival of an invader before the onset of disturbance could increase the impact of the environmental disturbance on bacterial diversity and structure. The Cold_T__0_/Cold_T__20_ treatment, on the other hand, impacted only slightly α-diversity estimates, mainly in soil D, compared to control microcosms. However no significant differences were found both at T20 and T40 between inoculated and non-inoculated microcosms. No major differences in terms of community composition were found between Cold_T__0_/Cold_T__20_ treated and control microcosms, whatever their inoculation status (Figure 6).

## Discussion

It is known that physico-chemical disturbances and invasion of new biological species are major processes affecting ecosystems (Ehrenfeld, 2010; Griffiths and Philippot, 2013; Calderon et al., 2018). However, it is unclear how the occurrence of these two processes simultaneously might affect the behavior of the invading species and the resident microbial community. In the present study, soil was selected as a model of heterogeneous habitat to investigate this phenomenon. Two types of soil characterized by distinct biotic environments and abiotic parameters (pH, mineral composition, resources available, water content…) were included in the study. In this setup, experiments carried out in sterile soil confirmed that these soils support the persistence of *L. monocytogenes* in the absence of microbiota. This is in agreement with previous studies showing that *L. monocytogenes* is able to use the resources available to grow in sterile soil during incubation at constant temperature (McLaughlin et al., 2011; Locatelli et al., 2013; Vivant et al., 2013b, 2015). Interestingly, in the present study, growth of *L. monocytogenes* was observed in sterile soil microcosms irrespective of other test conditions.

However, the presence of soil microbiota significantly influenced the behavior of *L. monocytogenes* in test environments. Indeed, the population of *L. monocytogenes* decreased in non-sterile soil microcosms under all conditions tested. This is consistent with the available literature which shows that the biotic environment has a major impact on the fate of *L. monocytogenes* in soil (McLaughlin et al., 2011; Locatelli et al., 2013; Vivant et al., 2013b, 2015). In particular, microbial diversity and community structure have been identified as key factors affecting microbial invaders in soil (van Elsas et al., 2012; Vivant et al., 2013a; Yao et al., 2014; Chapelle et al., 2016). Interestingly, loss of soil resident community diversity and increased resource availability were identified as the major parameters facilitating the subsequent invasion by *Pseudomonas fluorescens*, *Escherichia coli*, *Ralstonia solanacearum* and *Bacillus amyloliquefaciens*, once inoculated in heat-disturbed soil microcosms (Liu et al., 2012; Ma et al., 2015). Under non-sterile condition, habitat disturbance had a more significant influence on the fate of *L. monocytogenes* during invasion but results depended on the regimen of disturbances. A discrete rise of temperature improved the control of the invader and resulted in the decline of its population below the limit of detection. In contrast, reduction in temperature facilitated survival and resulted, in some instances, in successful invasion of the habitat. Because environmental disturbances are major drivers of soil microbial communities over time and impact ecosystem functioning (Griffiths and Philippot, 2013; Calderon et al., 2018), we investigated the changes in microbial diversity over time. Significant shifts of microbial communities were observed with changes dependent upon the type of disturbance. Our results suggest that, during habitat disturbances, variations of community diversity and structure modify the ability of the soil communities to control invasion. Indeed, abundance of several OTUs was significantly higher after treatment at 42°C in comparison to the other treatments. These heat-adaptive OTUs might be competitors of *L. monocytogenes* in the soil environment. Interestingly, members of *Actinobacteria* including *Streptomyces* are known to produce secondary metabolites with inhibitory activity toward *L. monocytogenes* (Undabarrena et al., 2016) while *Brevibacillus* produces bacteriocins active against *L. monocytogenes* (Sharma et al., 2014; Helfrich et al., 2018). Moreover, bacteriovorous bacteria from the *Haliangiaceae* class could participate to the predation of *L. monocytogenes* (Olanya and Lakshman, 2015). To our knowledge, no information is available on interactions or inhibitory activity of the spore-forming heat resistant *Alycyclobacillus*, *Balneimonas*, and *Kaistobacter* with *L. monocytogenes*.

Several OTUs were enriched in the Cold_T__0_/Cold_T__20_ treatment but their abundance was similar in control microcosms. This suggests that these OTUs displaying similar trends as *L. monocytogenes* might be affected by the temperature rise to 42°C rather than being resistant to freeze-thaw. It is therefore difficult to consider a possible role of these OTUs in facilitating the success of the invasive species in cold-disturbed habitats. To our knowledge no report is available on positive biotic interactions of these OTUs with *L. monocytogenes*.

The chronology of disturbances had a major impact on microbial diversity and community structure. The autochthonous microbial communities were resistant to freeze-thaw but temperature rise had major impacts upon the community structure. This confirms previous findings that the chronological order of disturbances needs to be taken into account (Calderon et al., 2018). Similarly, the fate of the invading species was different according to the type of soil. These two soils present abiotic characteristics that are diverse in terms of pH (Soil E pH of 7.15; soil D pH of 5.46) and texture (Soil E clay loamy soil; Soil D acidic loamy sandy soil). These parameters are known to affect fitness of *L. monocytogenes* in soil and lower survival was previously reported in sandy soils with pHs below 7 (Locatelli et al., 2013). More generally, pH and texture are major drivers of the structure of soil microbial communities (Lauber et al., 2008; Rousk et al., 2010). Not surprisingly, we observed significant differences in microbial diversity according to the type of soil. Indeed, complex interactions between components of the biotic environment and with the abiotic surroundings shape ecosystems (Faust and Raes, 2012; Cordero and Datta, 2016). Therefore, our results suggest that the impact of disturbances on the success of invasion is very much soil-dependent.

Invasion significantly impacted microbial diversity in our study. Arrival of allochthonous bacteria induced significant changes in the diversity and structure of the resident communities and our results confirm previous reports (Yao et al., 2014; Chapelle et al., 2016; Mallon et al., 2018). However, the shifts of diversity upon invasion depended on the regimen of disturbing factors. These bacterial communities were quite resistant to freeze-thaw disturbances, even during invasion, but heat disturbance had a more profound impact. Furthermore, the data suggest that invasion could have an even stronger effect on bacterial diversity when the environmental disturbance causes instability within the endogenous bacterial community.

In soil, bacteria are the most abundant active microorganisms but archaeal or fungal counterparts could be other factors influencing establishment of invasive species. To the best of our knowledge, information on their possible interactions with *L. monocytogenes* in soil is yet to be duly investigated. Altogether, our results demonstrate that environmental disturbances affect *L. monocytogenes* invasiveness patterns in soils. Intriguingly, persistence of *L. monocytogenes* was evidenced in a freeze-thaw disturbed soil displaying high microbial diversity but showing small community structure changes upon disturbance. Hence, community membership might be more important than diversity when it comes to preventing the establishment of an invading species. Two distinct characteristics seem to be required for an invading species to persist in a disturbed environment: (*i*) the invading species must be resistant to the disturbance and (*ii*) the disturbance must cause shifts in the composition of the autochthonous bacterial community in order to remove antagonists and/or competitors. Finally, we showed that the presence of an invading species might worsen the consequences of habitat disturbance, indicating that introduction of new microbial consortia in soil could destabilize the resident microbiota in the event of habitat disturbance.

## Data Availability Statement

The datasets generated for this study can be found in the SRA at NCBI under the accession number PRJNA506131.

## Author Contributions

AS, SG, and PP designed the experiments and wrote the manuscript. AC and DB performed the experiments. AS, SG, PP, LG, and DG analyzed the results.

## Conflict of Interest

The authors declare that the research was conducted in the absence of any commercial or financial relationships that could be construed as a potential conflict of interest.

## Supplementary Material

FIGURE S1 Assessment of soil inoculum pre-treatment on the dynamics of *L. monocytogenes* during invasion of control and disturbed soil microcosms. Inoculums were prepared by incubating *L. monocytogenes* for 1 week in sterile soil undergoing disturbance (cold, heat) or kept undisturbed at 20°C; 40 days old disturbed or undisturbed microcosms were then seeded with these conditioned soil inoculums. : Control inoculum;: Heat_T0_ inoculum;: Cold_T0_ inoculum.

FIGURE S2 Bacterial α-diversity in soil microcosms facing ongoing invasion and under a regimen of disturbances, estimated by evenness index.

FIGURE S3 OTUs displaying significantly higher abundance in the treatment ColdT0/ColdT20 than in the treatment HeatT0/HeatT20 applied to soil E microcosms. After 16SrDNA gene diversity analysis, abundance of detected OTUs was compared between treatments. Rarefaction of all samples was set at 3500 sequences. The frequency of each OTU was determined after dividing the number of reads by 3500. Significance of OTUs with higher abundance was assessed according to Tukey’s test (*P* < 0.05). Average and standard error of the mean are displayed.

FIGURE S4 OTUs displaying significantly higher abundance in the treatment HeatT0/HeatT20 than in the treatment ColdT0/ColdT20 applied to soil E microcosms. After 16SrDNA diversity analysis, abundance of detected OTUs was compared between treatments. Rarefaction of all samples was set at 3500 sequences. The frequency of each OTU was determined after dividing the number of reads by 3500. Significance of OTUs with higher abundance was assessed according to Tukey’s test (*P* < 0.05). Average and standard error of the mean are displayed.

FIGURE S5 Assessing the effect of *L. monocytogenes* inoculation on the initial bacterial diversity in soil E and soil D at T0. **(A)** Species richness estimated by the observed species index. **(B)** Evenness estimated by Simpson’s reciprocal index. **(C)** Phylogenetic diversity estimated by Faith’s phylogenetic diversity index. Results for inoculated and non-inoculated microcosms are shown in black and gray respectively.

TABLE S1 Summary of the significance of the differences of populations of *L. monocytogenes* between treatments and control during incubation in soil E and soil D microcosms (Tukey, *P* < 0.05) either sterilized by γ-irradiation or non-sterilized.
