# Supplementary figures and images for: Habitat Disturbances Modulate the Barrier Effect of Resident Soil Microbiota on Listeria monocytogenes Invasion Success
Source: Front Microbiol. 2020 May 28;11:927. doi: 10.3389/fmicb.2020.00927 (PMC7270165; doi:10.3389/fmicb.2020.00927)

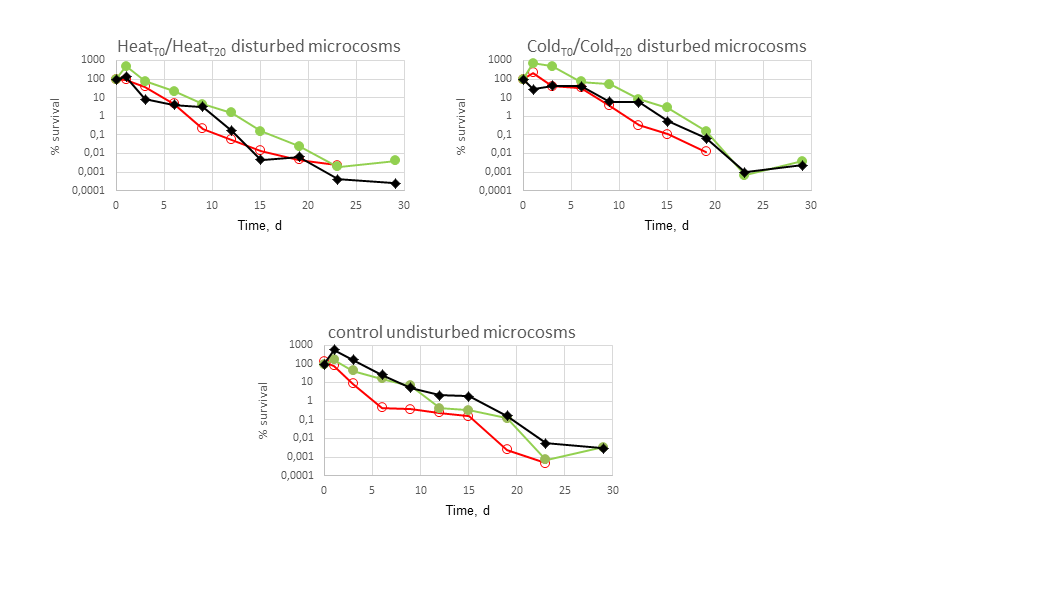

Supplement: FIGURE S1 — Assessment of soil inoculum pre-treatment on the dynamics of L. monocytogenes during invasion of control and disturbed soil microcosms. Inoculums were prepared by incubating L. monocytogenes for 1 week in sterile soil undergoing disturbance (cold, heat) or kept undisturbed at 20°C; 40 days old disturbed or undisturbed microcosms were then seeded with these conditioned soil inoculums. : Control inoculum;: HeatT0 inoculum;: ColdT0 inoculum. [file Image_1.tif]

Experiment 1

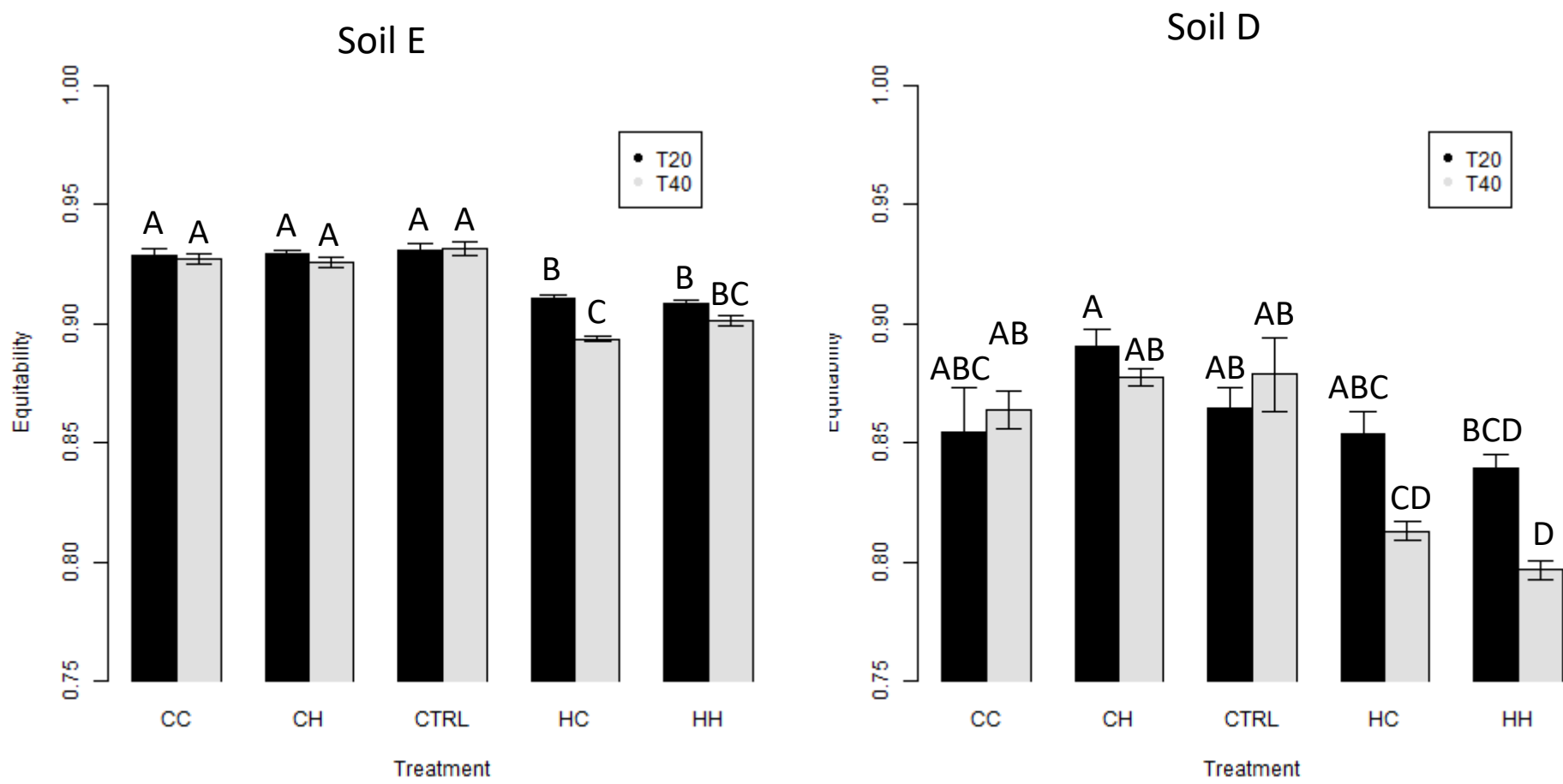

# Experiment 3

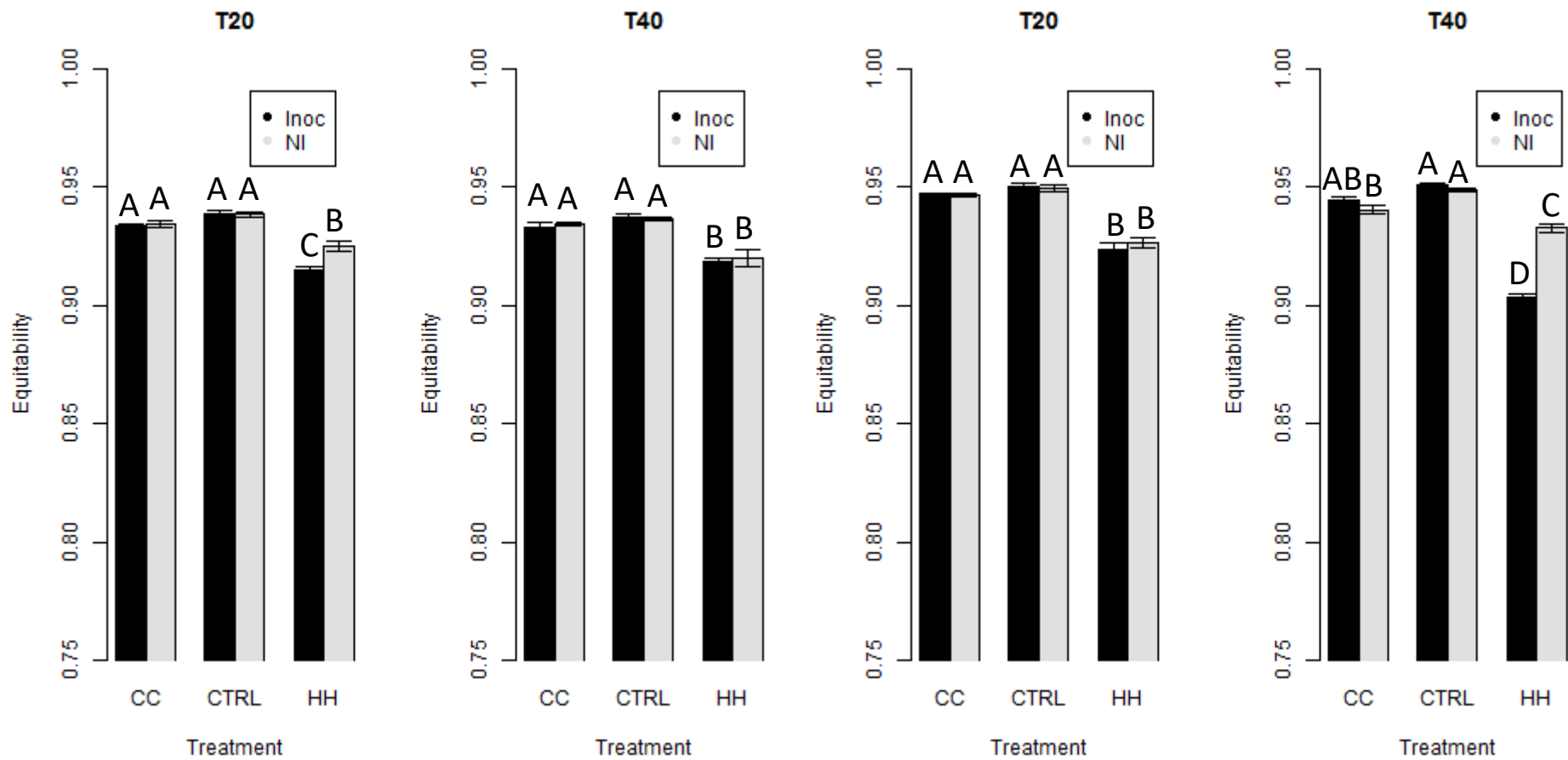

Supplement: FIGURE S2 — Bacterial α-diversity in soil microcosms facing ongoing invasion and under a regimen of disturbances, estimated by evenness index. [file Image_2.pdf]

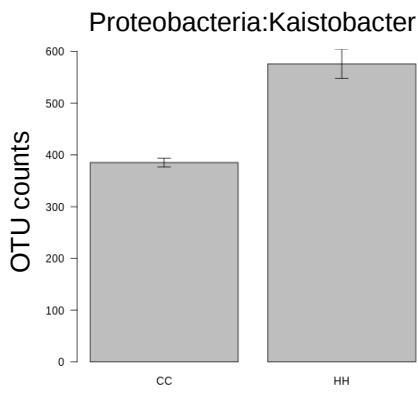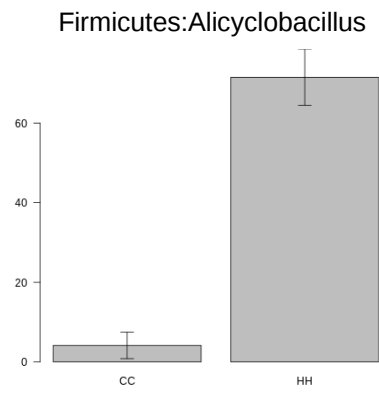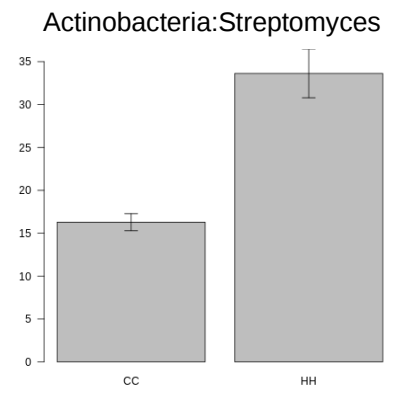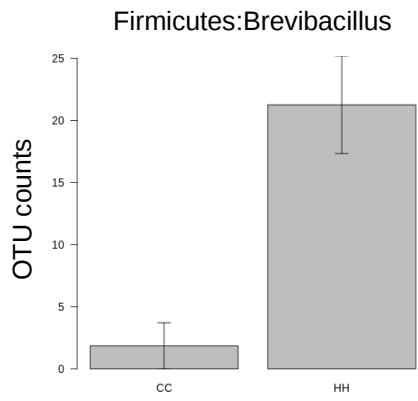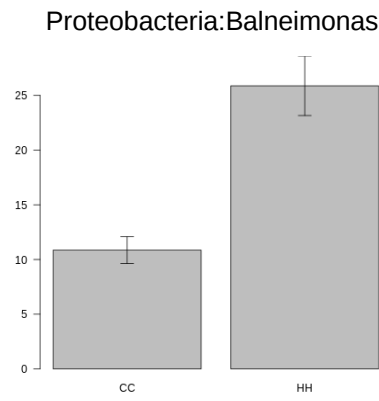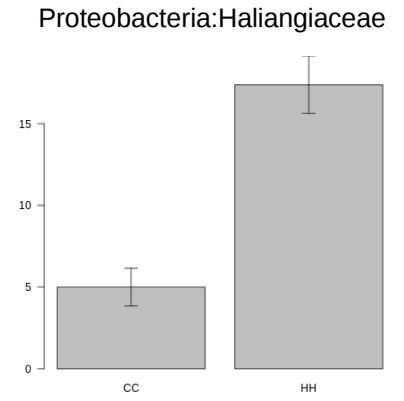

Supplement: FIGURE S4 — OTUs displaying significantly higher abundance in the treatment HeatT0/HeatT20 than in the treatment ColdT0/ColdT20 applied to soil E microcosms. After 16SrDNA diversity analysis, abundance of detected OTUs was compared between treatments. Rarefaction of all samples was set at 3500 sequences. The frequency of each OTU was determined after dividing the number of reads by 3500. Significance of OTUs with higher abundance was assessed according to Tukey’s test (P < 0.05). Average and standard error of the mean are displayed. [file Image_4.pdf]

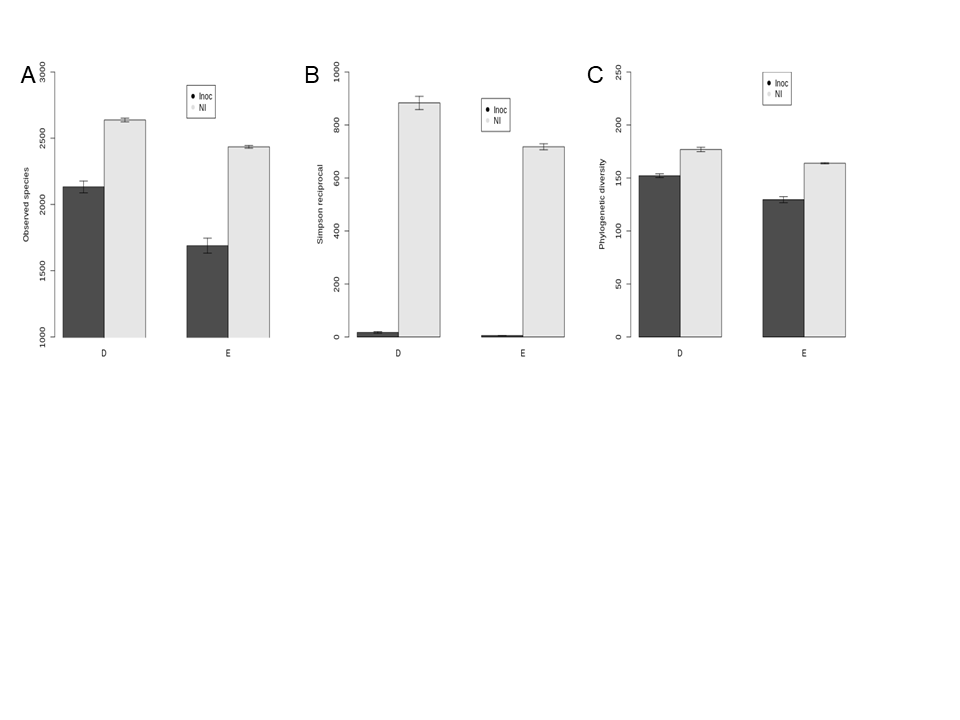

Supplement: FIGURE S5 — Assessing the effect of L. monocytogenes inoculation on the initial bacterial diversity in soil E and soil D at T0. (A) Species richness estimated by the observed species index. (B) Evenness estimated by Simpson’s reciprocal index. (C) Phylogenetic diversity estimated by Faith’s phylogenetic diversity index. Results for inoculated and non-inoculated microcosms are shown in black and gray respectively. [file Image_5.tif]
