# Supplementary material for: Habitat Disturbances Modulate the Barrier Effect of Resident Soil Microbiota on Listeria monocytogenes Invasion Success
Source: Front Microbiol. 2020 May 28;11:927. doi: 10.3389/fmicb.2020.00927 (PMC7270165; doi:10.3389/fmicb.2020.00927)
Supplement: FIGURE S3 — OTUs displaying significantly higher abundance in the treatment ColdT0/ColdT20 than in the treatment HeatT0/HeatT20 applied to soil E microcosms. After 16SrDNA gene diversity analysis, abundance of detected OTUs was compared between treatments. Rarefaction of all samples was set at 3500 sequences. The frequency of each OTU was determined after dividing the number of reads by 3500. Significance of OTUs with higher abundance was assessed according to Tukey’s test (P < 0.05). Average and standard error of the mean are displayed. [file Image_3.pdf]

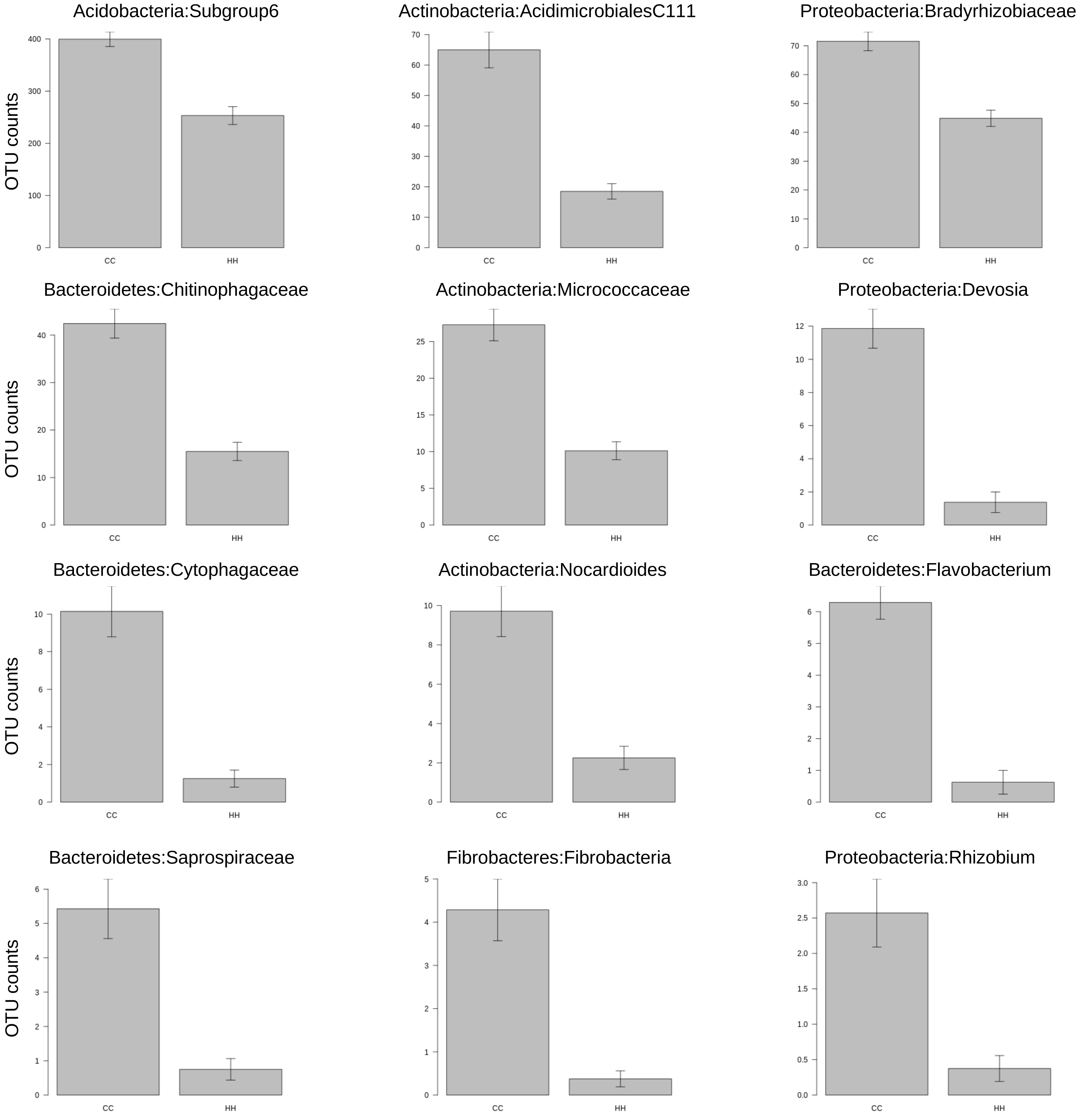
Acidobacteria:Subgroup6
Actinobacteria:AcidimicrobialesC111
Proteobacteria:Bradyrhizobiaceae
Bacteroidetes:Chitinophagaceae
Actinobacteria:Micrococcaceae
Proteobacteria:Devosia
Bacteroidetes:Cytophagaceae
Actinobacteria:Nocardioides
Bacteroidetes:Flavobacterium
Bacteroidetes:Saprospiraceae
Fibrobacteres:Fibrobacteria
Proteobacteria:Rhizobium
OTU counts
CC
HH
