# Supplementary material for: Habitat Disturbances Modulate the Barrier Effect of Resident Soil Microbiota on Listeria monocytogenes Invasion Success
Source: Front Microbiol. 2020 May 28;11:927. doi: 10.3389/fmicb.2020.00927 (PMC7270165; doi:10.3389/fmicb.2020.00927)
Supplement: TABLE S1 — Summary of the significance of the differences of populations of L. monocytogenes between treatments and control during incubation in soil E and soil D microcosms (Tukey, P < 0.05) either sterilized by γ-irradiation or non-sterilized. [file Table_1.pdf]

**SOIL E sterile**

| | | | | | | |
|---|---|---|---|---|---|---|
| 0 | Treatment | ESC | ES-20/-20 | ES-20/42 | ES42/-20 | ES42/42 |
| | Average | 7,675 | 7,750 | 7,725 | 7,725 | 7,475 |
| | Standard error | 155 | 155 | 155 | 155 | 155 |
| | Groups | A | A | A | A | A |

| | | | | | | |
|---|---|---|---|---|---|---|
| 2 | Treatment | ESC | ES-20/-20 | ES-20/42 | ES42/-20 | ES42/42 |
| | Average | 8,250 | 8,275 | 8,200 | 8,250 | 8,275 |
| | Standard error | 51 | 51 | 51 | 51 | 51 |
| | Groups | A | A | A | A | A |

| | | | | | | |
|---|---|---|---|---|---|---|
| 5 | Treatment | ESC | ES-20/-20 | ES-20/42 | ES42/-20 | ES42/42 |
| | Average | 8,475 | 8,500 | 8,400 | 7,975 | 7,975 |
| | Standard error | 52 | 52 | 52 | 52 | 52 |
| | Groups | A | A | A | | |
| | | | | | B | B |

| | | | | | | |
|---|---|---|---|---|---|---|
| 9 | Treatment | ESC | ES-20/-20 | ES-20/42 | ES42/-20 | ES42/42 |
| | Average | 8,400 | 8,375 | 8,375 | 7,850 | 7,900 |
| | Standard error | 66 | 66 | 66 | 66 | 66 |
| | Groups | A | A | A | | |
| | | | | | B | B |

| | | | | | | |
|---|---|---|---|---|---|---|
| 15 | Treatment | ESC | ES-20/-20 | ES-20/42 | ES42/-20 | ES42/42 |
| | Average | 8,475 | 8,475 | 8,400 | 7,725 | 7,725 |
| | Standard error | 70 | 70 | 70 | 70 | 70 |
| | Groups | A | A | A | | |
| | | | | | B | B |

| | | | | | | |
|---|---|---|---|---|---|---|
| 23 | Treatment | ESC | ES-20/-20 | ES-20/42 | ES42/-20 | ES42/42 |
| | Average | 8,250 | 8,150 | 7,850 | 7,550 | 7,025 |
| | Standard error | 74 | 74 | 74 | 74 | 74 |
| | Groups | A | A | | | |
| | | | B | B | | |
| | | | | C | C | |
| | | | | | | D |

| | | | | | | |
|---|---|---|---|---|---|---|
| 26 | Treatment | ESC | ES-20/-20 | ES42/-20 | ES-20/42 | ES42/42 |
| | Average | 8,300 | 8,200 | 7,650 | 7,250 | 6,850 |
| | Standard error | 70 | 70 | 70 | 70 | 70 |
| | Groups | A | A | | | |
| | | | | B | | |
| | | | | | C | |
| | | | | | | D |

| | | | | | | |
|---|---|---|---|---|---|---|
| 33 | Treatment | ESC | ES-20/-20 | ES42/-20 | ES-20/42 | ES42/42 |
| | Average | 8,325 | 8,200 | 7,725 | 7,325 | 7,175 |
| | Standard error | 107 | 107 | 107 | 107 | 107 |
| | Groups | A | A | | | |
| | | | | B | B | |
| | | | | | C | C |

| | | | | | | |
|---|---|---|---|---|---|---|
| 40 | Treatment | ESC | ES-20/-20 | ES42/-20 | ES-20/42 | ES42/42 |
| | Average | 8,375 | 8,175 | 7,525 | 7,475 | 6,850 |
| | Standard error | 153 | 153 | 153 | 153 | 153 |
| | Groups | A | A | | | |
| | | | B | B | | |
| | | | | C | C | |
| | | | | | D | D |

**SOIL D sterile**

| | | | | | | |
|---|---|---|---|---|---|---|
| 0 | Treatment | DSC | DS-20/-20 | DS-20/42 | DS42/-20 | DS42/42 |
| | Average | 7,625 | 7,675 | 7,800 | 7,675 | 7,900 |
| | Standard error | 120 | 120 | 120 | 120 | 120 |
| | Groups | A | A | A | A | A |

| | | | | | | |
|---|---|---|---|---|---|---|
| 2 | Treatment | DSC | DS-20/-20 | DS-20/42 | DS42/-20 | DS42/42 |
| | Average | 8,350 | 7,750 | 7,925 | 7,725 | 7,775 |
| | Standard error | 765 | 765 | 765 | 765 | 765 |
| | Groups | A | | | | |
| | | | B | B | B | B |

| | | | | | | |
|---|---|---|---|---|---|---|
| 5 | Treatment | DSC | DS-20/-20 | DS-20/42 | DS42/-20 | DS42/42 |
| | Average | 8,475 | 8,200 | 8,350 | 7,325 | 7,425 |
| | Standard error | 65 | 65 | 65 | 65 | 65 |
| | Groups | A | A | A | | |
| | | | | | B | B |

| | | | | | | |
|---|---|---|---|---|---|---|
| 9 | Treatment | DSC | DS-20/-20 | DS-20/42 | DS42/-20 | DS42/42 |
| | Average | 8,675 | 8,250 | 8,300 | 7,250 | 7,175 |
| | Standard error | 145 | 145 | 145 | 145 | 145 |
| | Groups | A | A | A | | |
| | | | | | B | B |

| | | | | | | |
|---|---|---|---|---|---|---|
| 15 | Treatment | DSC | DS-20/-20 | DS-20/42 | DS42/-20 | DS42/42 |
| | Average | 8,450 | 8,200 | 8,225 | 7,025 | 7,100 |
| | Standard error | 88 | 88 | 88 | 88 | 88 |
| | Groups | A | A | A | | |
| | | | | | B | B |

| | | | | | | |
|---|---|---|---|---|---|---|
| 26 | Treatment | DSC | DS-20/-20 | DS42/-20 | DS42/42 | DS-20/42 |
| | Average | 8,225 | 8,000 | 7,325 | 6,700 | 6,150 |
| | Standard error | 114 | 114 | 114 | 114 | 114 |
| | Groups | A | A | | | |
| | | | | B | | |
| | | | | | C | |
| | | | | | | D |

| | | | | | | |
|---|---|---|---|---|---|---|
| 33 | Treatment | DSC | DS-20/-20 | DS-20/42 | DS42/-20 | DS42/42 |
| | Average | 8,050 | 7,725 | 6,875 | 7,225 | 5,850 |
| | Standard error | 110 | 110 | 110 | 110 | 110 |
| | Groups | A | A | | | |
| | | | | B | B | |
| | | | | | | C |

| | | | | | | |
|---|---|---|---|---|---|---|
| 40 | Treatment | DSC | DS-20/-20 | DS-20/42 | DS42/-20 | DS42/42 |
| | Average | 8,075 | 7,500 | 7,050 | 7,350 | 6,725 |
| | Standard error | 160 | 160 | 160 | 160 | 160 |
| | Groups | A | A | | | |
| | | | B | B | B | |
| | | | | C | C | C |

**SOIL E non-sterile**

| | | | | | | |
|---|---|---|---|---|---|---|
| 0 | Treatment | EC | E-20/-20 | E-20/42 | E42/-20 | E42/42 |
| | Average | 8,075 | 8,475 | 8,880 | 8,525 | 8,650 |
| | Standard error | 335 | 335 | 335 | 335 | 335 |
| | Groups | A | A | A | A | A |

| | | | | | | |
|---|---|---|---|---|---|---|
| 2 | Treatment | EC | E-20/-20 | E-20/42 | E42/-20 | E42/42 |
| | Average | 7,225 | 8,350 | 8,300 | 4,225 | 4,400 |
| | Standard error | 104 | 104 | 104 | 104 | 104 |
| | | | A | A | | |
| | Groups | B | | | | |
| | | | | | C | C |

| | | | | | | |
|---|---|---|---|---|---|---|
| 5 | Treatment | E-20/-20 | E-20/42 | EC | E42/-20 | E42/42 |
| | Average | 7,525 | 7,350 | 5,900 | 2,750 | 2,200 |
| | Standard error | 105 | 105 | 105 | 105 | 105 |
| | | A | A | | | |
| | | | | B | | |
| | Groups | | | | C | |
| | | | | | | D |

| | | | | | | |
|---|---|---|---|---|---|---|
| 9 | Treatment | E-20/-20 | E-20/42 | EC | E42/-20 | E42/42 |
| | Average | 4,750 | 4,725 | 3,200 | 1,900 | 2,175 |
| | Standard error | 144 | 144 | 144 | 144 | 144 |
| | | A | A | | | |
| | Groups | | | B | | |
| | | | | | C | C |

| | | | | | | |
|---|---|---|---|---|---|---|
| 15 | Treatment | E-20/-20 | E-20/42 | EC | E42/-20 | E42/42 |
| | Average | 3,700 | 3,875 | 3,000 | 2,225 | 2,375 |
| | Standard error | 102 | 102 | 102 | 102 | 102 |
| | | A | A | | | |
| | Groups | | | B | | |
| | | | | | C | C |

| | | | | | | |
|---|---|---|---|---|---|---|
| 23 | Treatment | E-20/-20 | E-20/42 | EC | E42/-20 | E42/42 |
| | Average | 2,975 | 2,825 | 2,075 | 1,200 | 1,350 |
| | Standard error | 81 | 81 | 81 | 81 | 81 |
| | | A | A | | | |
| | Groups | | | B | | |
| | | | | | C | C |

| | | | | | | |
|---|---|---|---|---|---|---|
| 26 | Treatment | E-20/-20 | EC | E-20/42 | E42/-20 | E42/42 |
| | Average | 2,850 | 1,875 | 1,550 | 1,350 | 1,425 |
| | Standard error | 119 | 119 | 119 | 119 | 119 |
| | | A | | | | |
| | Groups | | B | B | | B |
| | | | | C | C | C |

| | | | | | | |
|---|---|---|---|---|---|---|
| 33 | Treatment | E-20/-20 | E-20/42 | E42/-20 | EC | E42/42 |
| | Average | 1,725 | 0,750 | 0,600 | ND | ND |
| | Standard error | 263 | 263 | 263 | 263 | 263 |
| | Groups | A | A | A | | |
| | | | B | B | B | B |

| | | | | | | |
|---|---|---|---|---|---|---|
| 40 | Treatment | E-20/-20 | EC | E-20/42 | E42/-20 | E42/42 |
| | Average | 1,625 | ND | ND | ND | ND |
| | Standard error | 42 | 42 | 42 | 42 | 42 |
| | Groups | A | B | B | B | B |

**SOIL D non-sterile**

| | | | | | | |
|---|---|---|---|---|---|---|
| 0 | Treatment | DC | D-20/-20 | D-20/42 | D42/-20 | D42/42 |
| | Average | 8,600 | 8,750 | 8,725 | 8,700 | 8,750 |
| | Standard error | 74 | 74 | 74 | 74 | 74 |
| | Groups | A | A | A | A | A |

| | | | | | | |
|---|---|---|---|---|---|---|
| 2 | Treatment | D-20/-20 | D-20/42 | DC | D42/-20 | D42/42 |
| | Average | 8,425 | 8,500 | 5,250 | 3,625 | 3,475 |
| | Standard error | 231 | 231 | 231 | 231 | 231 |
| | Groups | A | A | B | C | C |

| | | | | | | |
|---|---|---|---|---|---|---|
| 5 | Treatment | D-20/-20 | D-20/42 | DC | D42/-20 | D42/42 |
| | Average | 6,400 | 6,500 | 5,075 | 2,800 | 2,500 |
| | Standard error | 144 | 144 | 144 | 144 | 144 |
| | Groups | A | A | B | C | C |

| | | | | | | |
|---|---|---|---|---|---|---|
| 9 | Treatment | DC | D-20/-20 | D-20/42 | D42/-20 | D42/42 |
| | Average | 3,475 | 3,325 | 3,275 | 1,425 | 1,475 |
| | Standard error | 235 | 235 | 235 | 235 | 235 |
| | Groups | A | A | A | B | B |

| | | | | | | |
|---|---|---|---|---|---|---|
| 15 | Treatment | D-20/42 | D-20/-20 | DC | D42/-20 | D42/42 |
| | Average | 1,750 | 1,600 | 1,275 | 1,275 | 1,475 |
| | Standard error | 83 | 83 | 83 | 83 | 83 |
| | Groups | A | A<br>B | B | B | B |
